# Phenotypic Heterogeneity in ORAI-1-Associated Congenital Myopathy

**DOI:** 10.1055/s-0044-1790245

**Published:** 2024-09-05

**Authors:** Dipti Baskar, Seena Vengalil, Kiran Polavarapu, Veeramani Preethish-Kumar, Gautham Arunachal, Ramya Sukrutha, Mainak Bardhan, Akshata Huddar, Gopikrishnan Unnikrishnan, Girish Baburao Kulkarni, Yasha T. Chickabasaviah, Rashmi Santhosh Kumar, Atchayaram Nalini, Saraswati Nashi

**Affiliations:** 1 Department of Neurology, National Institute of Mental Health and Neuro Sciences, Bengaluru, Karnataka, India; 2 Division of Neurology, Children's Hospital of Eastern Ontario Research Institute, The Ottawa Hospital, University of Ottawa, Ottawa, Canada; 3 Department of Neurology, Morriston Hospital, Swansea Bay University Health Board, Swansea, United Kingdom; 4 Department of Human Genetics, National Institute of Mental Health and Neuro Sciences, Bengaluru, Karnataka, India; 5 Department of Neuropathology, National Institute of Mental Health and Neuro Sciences, Bengaluru, Karnataka, India

**Keywords:** ORAI-1, ophthalmoparesis, congenital myopathy, congenital fiber-type disproportion

## Abstract

**Introduction** ORAI-1 is a plasma membrane calcium release-activated calcium channel that plays a crucial role in the excitation–contraction of skeletal muscles. Loss-of-function mutations of *ORAI-1* cause severe combined immunodeficiency, nonprogressive muscle hypotonia, and anhidrotic ectodermal dysplasia. Autosomal dominant gain-of-function mutation causes Stormorken's syndrome, which includes tubular aggregate myopathy along with bleeding diathesis.

**Methods** This is a description of a genetically confirmed case of ORAI-1-associated myopathy with clinical, histopathological, and imaging characteristics and a detailed literature review.

**Results** We report an 18-year-old woman who presented with 2-and-a-half year history of slowly progressive proximal lower limb weakness and ophthalmoparesis. Her serum creatine kinase levels were normal. Magnetic resonance imaging of the muscle showed predominant fatty infiltration of the glutei and quadriceps femoris. Histopathological analysis of muscle biopsy was suggestive of congenital fiber-type disproportion (CFTD). Clinical exome sequencing showed novel homozygous nonsense pathogenic variant NC_000012.12 (NM_032790.3): c.205G > T (p.Glu69Ter) in *ORAI-1* gene.

**Conclusion** This report expands the phenotypic spectrum of ORAI-1-related myopathy to include congenital myopathy—CFTD with ophthalmoparesis, a novel manifestation.

## Introduction

Calcium ion (Ca ^2+^ ) is an important signaling molecule for regulating many cellular processes, especially skeletal muscle contraction. A range of Ca ^2+^ channels and transporters tightly regulate cytosolic Ca ^2+^ concentration for the excitation-contraction coupling of skeletal muscles. 1 Store-operated Ca ^2+^ entry (SOCE) is an important pathway involved in muscle contraction, which in turn is mediated by two protein families: stromal interaction molecules (STIMs) 1 and 2 of endoplasmic reticulum and ORAI calcium release-activated calcium modulator proteins forming Ca ^2+^ release-activated calcium (CRAC) channel in the plasma membrane both of which regulate the SOCE pathway. 2 3 ORAI-1 is the most important component of the CRAC channel, forming a hexameric Ca ^2+^ -binding channel. A variety of null and loss-of-function (LoF) variations in *ORAI-1* and *STIM1* results in a distinct entity called “CRAC channelopathy,” a multisystem disorder characterized by severe combined immunodeficiency (SCID) with recurrent infections, autoimmune hemolytic anemia/thrombocytopenia, anhidrotic ectodermal dysplasia, and nonprogressive muscle hypotonia. Gain-of-function (GoF) mutation causes Stormorken's syndrome and York platelet syndrome, including bleeding diathesis, miosis, and tubular aggregate myopathy. 4 5 Here, we report for the first time from India a unique homozygous *ORAI-1* variation presenting clinically as congenital myopathy and histologically as congenital fiber-type disproportion (CFTD).

## Methods

This retrospective analysis was done at a quaternary neurology center in southern India. Demographic and detailed clinical assessment data were recorded. Magnetic resonance imaging (MRI) of the muscle with T1-weighted and T2/short tau inversion recovery sequences was done to assess the fatty infiltration by modified Mercuri score and edema by Stramare score. 6 7 Histopathological analysis of the muscle biopsy from quadriceps femoris was done using hematoxylin and eosin, succinic dehydrogenase, and ATPase, and ultrastructural analysis was performed. Genetic analysis was done by clinical exome sequencing with coverage of 8332 clinical exome assay genes (critical genes analyzed are given in Supplementary Table S1 ). DNA extracted from blood was used to perform targeted gene capture using a custom capture kit. The libraries were sequenced to mean >80 to 100X coverage on Illumina sequencing platform. The Genome Analysis Toolkit best practices framework for identifying variants in the sample using Sentieon (v201808.01) were followed. 8 The sequences obtained were aligned to the human reference genome (GRCh37/hg19) using Sentieon aligner and analyzed using Sentieon to remove duplicates, recalibration, and realign indels. Sentieon haplotypecaller was used to identify variants that were relevant to the clinical indication. Gene annotation of the variants was performed using the VEP program 9 against the Ensemble release 91 human gene model. 10 Common variants are filtered based on allele frequency in 1000 Genome Phase 3, ExAC (v1.0), gnomAD (v2.1), EVS, dbSNP (v151), 1000 Japanese Genome, and our internal Indian population database. The nonsynonymous variants effect is calculated using multiple algorithms such as PolyPhen-2, SIFT, MutationTaster2, and LRT. Only nonsynonymous and splice site variants found in the clinical exome panel consisting of 8,332 genes were used for clinical interpretation. The pathogenic variants are presented according to standards of American College of Medical Genetics and Genomics (ACMG). 11 For the Sanger sequencing and segregation analysis of the second variant of unknown significance, polymerase chain reaction (PCR) was performed using primers designed to amplify the genomic region spanning the targeted variant in the exon. PCR products were confirmed by gel electrophoresis followed by treatment with ExoSAP to digest unutilized primers. The amplicons were subjected to cycle sequencing PCR using BigDye Terminator v3.1 kit as per manufacturer instructions. Sanger sequencing was performed on SeqStudio Genetic Analyzer. The variant at the targeted locus was ascertained by visual inspection of the electropherogram, as well as comparing it with the reference sequence. Informed consent for clinical details and patient figure publication was obtained from the patient.

## Results

An 18-year-old woman born to consanguineous parents presented with a 2-and-a-half-year history of pain in the thighs and arms. Six months later, she developed progressive lower limb proximal muscle weakness and in the next year noticed difficulty in walking. There was no distal lower limb or upper limb weakness, ocular symptoms, or bulbar weakness. She was noticed to be a slow runner since childhood. She also had a squint, which was noticed by the parents. She did not have recurrent infections. There was no significant family history.

On examination, she had a slender habitus, an elongated long face, and ophthalmoparesis. Hypotonia of lower limbs was noted. According to the modified Medical Research Council grading, muscle strength of the neck and upper limbs was grade 4. Bilateral hip flexion, extension, adduction, abduction, knee flexion, and extension were grade 3; ankle dorsiflexion and plantar flexion were grade 4. Tendon reflexes and sensory examination were normal. She had a waddling gait.

**Fig. 1:**
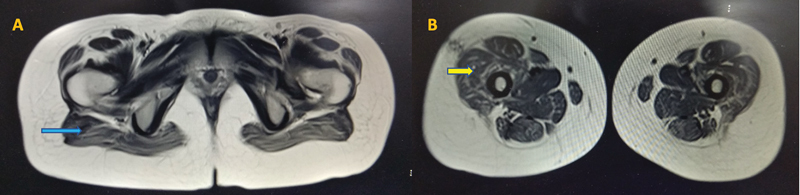
Muscle MRI of the patient: (A) and (B) show muscle MRI–T2 sequences that show fatty infiltration with atrophy of the glutei (blue arrow) and quadriceps (yellow arrow). MRI, magnetic resonance imaging.

**Fig. 2:**
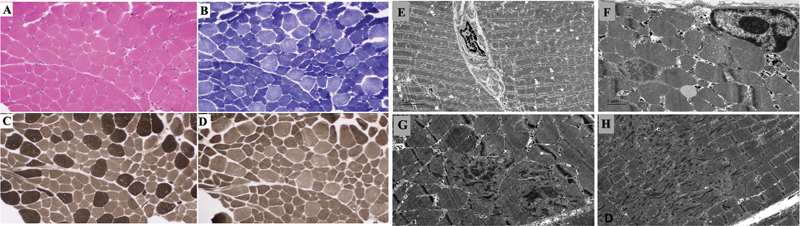
Histopathological images of muscle biopsy of the patient: (A) The hematoxylin and eosin–stained section shows an admixture of larger myofibers with several smaller fibers. (B), (C), and (D) reveal the more numerous hypotrophic, type 1 fibers that are dark on succinic dehydrogenase stain (B), light on ATPase 9.4 (C), and dark on ATPase ph4.6. Note somewhat fairly uniform size of the type 1 fibers. Ultrastructure of muscle biopsy: (E) Low power view of two adjoining myofibers separated by endomysial connective tissue. (F) A portion of a muscle fiber showing a subsarcolemmal myonucleus and focal disorganization of a myofilament (red star). No abnormal subsarcolemmal tubular aggregates or other deposits are seen. (G) Transverse and (H) longitudinal sections displaying disorganization of myofilamentous architecture with Z-band streaming and aggregation (red star). (Magnification included.)

Her investigations showed normal complete blood count, serum creatine kinase, and renal and liver functions. Muscle MRI of lower limbs showed fatty infiltration with Mercuri scale grade 3 (washed-out appearance) in glutei and grade 2b (late moth-eaten appearance) in quadriceps femoris muscles ( Fig. 1 ). Histopathological analysis of muscle biopsy from quadriceps femoris showed type 1 fiber predominance which were hypotrophic with the absence of rods or cores in electron microscopy suggestive of CFTD ( Fig. 2A–D ). On electron microscopy, a few fibers with focal Z-band disorganization and aggregation of Z-band material resembling minicore-like areas were observed. There was an absence of rods, cores, and tubular aggregates ( Fig. 2E–H ).

**Fig. 3:**
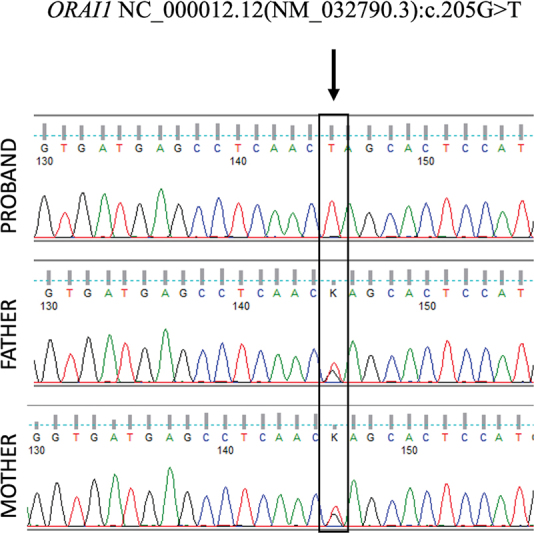
Electropherogram of patient and parents showing homozygous variant c.205G > T (p.Glu69Ter) in *ORAI-1* gene.

A novel pathogenic homozygous premature termination codon in exon 1 of the *ORAI-1* gene NC_000012.12 (NM_032790.3): c.205G > T; NP_116179.2: p.Glu69Ter (ACMG criteria: PM2 PVS1 PP4 as per ACMG guidelines 11 ) was detected at a depth of 228x ( Fig. 3 ). This variant is novel (not reported in any individuals) in gnomAD and has also not been reported in the 1000 Genomes, ExAC, TOPMed, and our internal databases. This variant is predicted to cause loss of normal protein function through protein truncation. This is a stop-gained variant that occurs in an exon of *ORAI-1* upstream of where nonsense-mediated decay is predicted to occur. There are seven downstream pathogenic LoF variants, with the furthest variant being 200 residues downstream of this variant. This indicates that the region is critical to protein function. The p.Glu69Ter variant is an LoF variant in the gene *ORAI-1* , which is intolerant of LoF variants, as indicated by the presence of existing pathogenic LoF variants NP_116179.2:p.S49Pfs*52 and NP_116179.2:p.S71Hfs*17. Sanger validation and segregation analysis revealed the variant to be segregating in a heterozygous state in the asymptomatic parents. In addition, the proband's clinical phenotype matches that of the disorder caused by pathogenic variants in the *ORAI-1* gene. For these reasons, this variant has been classified as pathogenic.

## Discussion

The clinical features described in our patient were suggestive of a slowly progressive congenital myopathy, which revealed features of CFTD on muscle biopsy with ophthalmoparesis. Congenital myopathies are clinically, histologically, and genetically heterogeneous structural disorders of skeletal muscles. The classification of congenital myopathies is primarily based on muscle biopsy features and, recently, on genetic characterization. 12 CFTD is a type of congenital myopathy defined histologically by disproportionately smaller and numerous type 1 fibers compared with type 2 fibers in the absence of other abnormal features such as rods or cores. It has an onset of symptoms mostly in the first decade of life with a very slowly progressive course. Clinically affected children present with mild facial dysmorphism, delayed motor milestones, muscle hypotonia, absent tendon reflexes, and limb-girdle type of muscle weakness. 13 These characteristic features were seen in our patient. Interestingly, our patient also had ophthalmoparesis, which has been previously described and considered specific for RYR-1-associated CFTD. 14 Several genotypic variations have been described in CFTD. The most common is an autosomal dominant or recessive mutation in α tropomyosin-3 ( *TPM-3* ), 15 followed by ryanodine receptor ( *RYR-1* ) variation. 14 Other variations described include selenoprotein-N ( *SEPN-1* ), α-actin-1 ( *ACTA-1* ), myosin heavy chain-7 ( *MYH-7* ), and tropomyosin-2 ( *TPM-2* ). 12

Most of the LoF *ORAI-1* variations are due to frameshifts, splice site defects, or substitutions involving the transmembrane domains and occur as null variations that interfere with protein expression, folding of α-helical transmembrane domains, and protein stability. 4 SCID-like immunodeficiency is common and noted in most patients with ORAI-1 LoF variation, characterized by recurrent and life-threatening viral, bacterial, and fungal infections. 4 5 However, the presence of autoimmunity is less common with ORAI-1 than with *STIM1* variations. 5 16 The absence of obvious manifestations of nonmuscular features of CRAC channelopathy, such as ectodermal defects and immune abnormalities, can be noted in patients with heterozygous LoF *ORAI-1* variants owing to retained residual SOCE function. 4 17 However, it is interesting that patient in the current study presented with a pure muscular phenotype despite a homozygous *ORAI-1* variant which has not been reported previously.

Myopathies associated with *ORAI-1* variations have been described with both LoF and GoF mutations. The nonprogressive muscle hypotonia of LoF mutation of *ORAI-1* is characterized by poor muscle strength and endurance since infancy, including respiratory muscle weakness and hypernasal voice. 5 16 Frequently, muscle hypotonia is associated with iris hypoplasia and mydriasis. 5 18 Muscle biopsy shows nonspecific findings such as atrophy of type 2 fibers with a predominance of type 1 fibers. 5 To date, about eight different LoF mutations have been described in *ORAI-1* . 19 Ophthalmoparesis reflecting the lack of concerted eye muscle contraction due to a defective SOCE pathway has been described in the GoF variation of *ORAI-1* . 20 21 No previous reports of LoF ORAI-1 variants are associated with CFTD and ophthalmoparesis, as noted in our patient ( Table 1 ).

**Table 1:** Comparison of previous studies on ORAI-1 myopathy

S. no.	Authors (year of study)	No. of patients	Age at onset	Clinical presentation				Muscle MRI	Muscle biopsy	Genotypic features
				Infections	Autoimmunity	Myopathy	Others			
1	Lacruz and Feske (2015) 4	1	<1	+	+			−	−	c.271C > T (p.Arg91Trp)
2	McCarl et al (2009) 5	6	<1	+1/2 + +	− + −	Congenital muscular hypotonia in all	Ectodermal anomalies (all three genotypes), mydriasis	–	Variation in muscle fiber size with a predominance of type 1 fibers and atrophic type 2 fibers (c.271C > T)	c.271C > T (p.Arg91Trp) (2) p.Ala88SerfsX25 (1) c.308C > A (p. Ala103Glu)/c.581T > C (p. Leu194Pro) (1) Not done in two
3	Maul-Pavicic et al (2011) 22	2	<1	+	+	Congenital muscular hypotonia	Ectodermal anomalies	–	–	c.271C > T (p.Arg91Trp)
4	Chou et al (2015) 23	1	<1	+	+	Congenital muscular hypotonia	–	–	–	c.493_494insC (p.H165Pfs)
5	Lian et al (2018) 24	4	<1	+	+ Absent (p.V181SfsX8)	Congenital muscular hypotonia	Ectodermal anomalies	–	Predominance of type 1 fibers and atrophic type 2 fibers (p.V181SfsX8)	p.V181SfsX8, c.581T > C (p. Leu194Pro), c.292G > C (p. Gly98Arg)
6	Our study (2022)	1	15–16	–	–	CFTD and ophthalmoparesis	–	Fatty infiltration of glutei and quadriceps femoris	CFTD—type 1 fiber predominance which was hypotrophic with absence of rods or cores in electron microscopy	c.205G > T (p.Glu69Ter)

Abbreviations: CFTD, congenital fiber-type disproportion; MRI, magnetic resonance imaging.

## Conclusion

We present an extremely rare case of slowly progressive myopathy and ophthalmoparesis with CFTD morphology on histology due to *ORAI-1* variation. Though *ORAI-1* is expressed in many tissues, our patient manifested a limited phenotype of only congenital myopathy. Further functional studies are required to identify the modulators of disease expression and reveal new therapeutic avenues. The current case expands the clinical and histological spectrum of ORAI-1-associated disorders.
