# Supplementary material for: Phenotypic Heterogeneity in ORAI-1-Associated Congenital Myopathy
Source: Glob Med Genet. 2024 Sep 5;11(4):297–303. doi: 10.1055/s-0044-1790245 (PMC11377103; doi:10.1055/s-0044-1790245)
Supplement: Supplementary file 1 — Supplementary Material [file 10-1055-s-0044-1790245-s2400066.pdf]

Supplementary Table S1 Clinical exome assay—critical genes covered

Gene	Percentage of coding region covered	Gene	Percentage of coding region covered	Gene	Percentage of coding region covered
ACTA1	100.00	*ADCY6*	100.00	*ADGRG6*	100.00
AGRN	100.00	*ALG14*	100.00	*ALG2*	100.00
ANO5	100.00	*B3GALNT2*	93.30	*B4GAT1*	100.00
BAG3	100.00	*BIN1*	100.00	*BVES*	100.00
CAPN3	100.00	*CAV3*	100.00	*CCDC78*	100.00
CFL2	100.00	*CHAT*	100.00	*CHKB*	100.00
CHRNA1	100.00	*CHRNB1*	100.00	*CHRND*	100.00
CHRNE	100.00	*CHRNG*	100.00	*CHST14*	100.00
CNTN1	100.00	*CNTNAP1*	100.00	*COL12A1*	100.00
COL13A1	100.00	*COL6A1*	100.00	*COL6A2*	100.00
COL6A3	100.00	*COLQ*	100.00	*CRYAB*	100.00
DAG1	100.00	*DES*	100.00	*DMD*	100.00
DNAJB6	100.00	*DNM2*	100.00	*DOK7*	100.00
DPAGT1	100.00	*DYSF*	100.00	*ECEL1*	100.00
EMD	100.00	*ERBB3*	100.00	*FBN2*	100.00
FHL1	100.00	*FKRP*	100.00	*FKTN*	100.00
FLNC	100.00	*GFPT1*	100.00	*GLE1*	100.00
GMPPB	100.00	*GNE*	100.00	*HNRNPA2B1*	100.00
HNRNPDL	100.00	*ISCU*	100.00	*ISPD*	100.00
ITGA7	100.00	*KBTBD13*	100.00	*KLHL40*	100.00
KLHL41	100.00	*LAMA2*	100.00	*LAMB2*	100.00
LAMP2	100.00	*LARGE*	100.00	*LDB3*	100.00
LIMS2	100.00	*LMNA*	100.00	*LMOD3*	100.00
LRP4	100.00	*MEGF10*	100.00	*MTM1*	100.00
MUSK	100.00	*MYBPC1*	100.00	*MYF6*	100.00
MYH2	100.00	*MYH3*	100.00	*MYH7*	100.00
MYH8	100.00	*MYOT*	100.00	*NALCN*	100.00
NEB	90.30	*ORAI1*	100.00	*PABPN1*	100.00
PI4KA	100.00	*PIEZO2*	100.00	*PIP5K1C*	100.00
PLEC	100.00	*POGLUT1*	100.00	*POMGNT1*	100.00
POMGNT2	100.00	*POMK*	100.00	*POMT1*	100.00
POMT2	100.00	*RAPSN*	100.00	*RYR1*	100.00
SCN4A	100.00	*SEPN1*	84.53	*SGCA*	100.00
SGCB	100.00	*SGCD*	100.00	*SGCG*	100.00
SLC18A3	100.00	*SLC35A3*	100.00	*SMCHD1*	100.00
SNAP25	100.00	*SPEG*	100.00	*SYNE1*	100.00
SYNE2	100.00	*SYT2*	100.00	*TCAP*	100.00
TMEM43	100.00	*TMEM5*	100.00	*TNNI2*	100.00
TNNT1	100.00	*TNNT3*	100.00	*TNPO3*	100.00
TOR1AIP1	100.00	*TPM2*	100.00	*TPM3*	100.00
TRAPPC11	100.00	*TRIM32*	100.00	*TTN*	99.59
UBA1	100.00	*VCP*	100.00	*VIPAS39*	100.00
VPS33B	100.00	*ZBTB42*	100.00		

 © 2024. The Author(s).
